# An Unusual Presentation of Left Ventricular Non-compaction Cardiomyopathy in a Female Patient With Sudden Cardiac Arrest: A Case Report

**DOI:** 10.7759/cureus.30830

**Published:** 2022-10-29

**Authors:** Tasniem Tasha, Ebubechukwu Ezeh, Nishantkumar Sonani

**Affiliations:** 1 Internal Medicine, Rajshahi Medical College, Rajshahi, BGD; 2 Internal Medicine, Marshall University, Joan C. Edwards School of Medicine, Huntington, USA; 3 Cardiology, Heart and Vascular Institute, Dearborn, USA

**Keywords:** implantable cardiac defibrillator (icd), arrhythmia, cardiomyopathy, left ventricular non compaction, sudden cardiac arrest

## Abstract

Left ventricular non-compaction (LVNC), a kind of cardiomyopathy, is characterized by excessive and prominent trabeculations in the mature left ventricle (LV). LVNC has been defined as the heart's developmental failure to fully form the compact myocardium during the latter stages of cardiac development. Clinical features vary from asymptomatic to symptomatic individuals with gradual loss of heart function, heart failure, thromboembolic events, arrhythmias, and sudden cardiac death are all possible outcomes. We describe a case of a 39-year-old Caucasian female who presented with a sudden cardiac arrest that was later attributed to LVNC. To the best of our knowledge, only a few occurrences are found in the literature where female patients with LVNC were presented with sudden cardiac arrest.

## Introduction

Left ventricular non-compaction (LVNC) cardiomyopathy is a rare congenital abnormality that describes a left ventricle (LV) wall anatomy characterized by three key morphologic elements, i.e., prominent trabeculae, a thin compacted layer, and intertrabecular recesses. It occurs due to the abnormal embryogenesis of the myocardium. Both left and right ventricles may be affected by non-compaction cardiomyopathy. Echocardiographic evidence of LVNC, seen in about 0.045% to 0.26% of adult patients and 0.01% to 1.3% of the general population, has a male preponderance [1]. Familial association of LVNC tends to range between 18% and 33% [2]. Clinical features are variable and range from asymptomatic to symptomatic patients with progressive deterioration in cardiac function. It can result in heart failure (HF), thromboembolic events, arrhythmias, and sudden cardiac death [3]. Though the evidence is limited in the literature, the presence of cardiac arrest and seizures in LVNC patients has been associated with high mortality. We describe an interesting case in which a 39-year-old female patient experienced sudden cardiac arrest that was later attributed to LVNC.

## Case presentation

A 39-year-old Caucasian female with a past medical history of chronic obstructive pulmonary disease (COPD), substance abuse, and obesity presented to the emergency department following a witnessed out-of-hospital cardiac arrest. According to the reports, the patient was at her friend’s birthday party, during which she suddenly experienced witnessed tonic-clonic seizure-like activities involving her bilateral upper extremities. Afterward, she was pulseless and reportedly went into cardiopulmonary collapse. Before the arrival of the Emergency Medical Services (EMS), bystanders provided cardiopulmonary resuscitation. She also received a total of naloxone 6mg without response. Upon the arrival of EMS 10 minutes later, defibrillator pads were placed and revealed pulseless ventricular fibrillation. One shock was delivered with the return of spontaneous circulation. She was treated using American Heart Association advanced cardiac life support protocol for ventricular fibrillation. After 10 minutes of cardiopulmonary resuscitation, drug protocol, and one defibrillator attempt, the patient returned to sinus rhythm and was intubated for airway protection on the route to the Emergency Department (ED).

Upon arrival at the hospital, the patient was noted to have a pulse of 99, blood pressure of 108/86, and saturation of 99%. Electrocardiogram (EKG) showed normal sinus rhythm with QTc 480ms. Next, she was taken to the resuscitation bay, and ventilatory support was provided. The patient was loaded with levetiracetam, and IV fluid was given. Initial lab findings are reported in Table 1.

**Table 1:** Laboratory data on initial presentation WBC= White Blood Count, BUN= Blood Uria Nitrogen, INR= International Normalised Ratio, ALT= Alanine Aminotransferase

Serum	Patient	Reference
WBC	15.1	4.5 to 11.0 × 109/L
Hemoglobin	13.2	12.1 to 15.1 g/dL
Sodium	132	136–146 (mmol/L)
potassium	4.1	3.5–5.5 (mmol/L)
CO2	12	23 to 29 (mEq/L)
Anion gap	17	4 to 12 mmol/L
BUN	7	6–20 (mg/dL)
Creatinine	1	0.4–12 (mg/dL)
Calcium	8.8	8.4–10.3 (mg/dL)
Phosphorus	5.4	2.5–5.0(mg/dL)
INR	1.01	< 1.1
ALT	54	0–31(U/L)
ALT	62	10–35(U/L)
Magnesium	2.0	1.9–2.7(mg/dL)
Glucose	95	70–99(mg/dL)
Bilirubin	0.5	0.0–1.2(mg/dL)
Troponin	4	0.0–0.15(ng/dL)

Her basic metabolic panel (BMP) revealed mild hyponatremia of 132 mmol/L (normal 136-146 mmol/L), bicarbonate 12 mEq/L (Normal 23 to 29 mEq/L), anion gap 17, lactate 5, and troponin 4. The CBC indicated leukocytosis (predominantly neutrophilic) to 15 macrocytic MCV. COVID-19 PCR result was negative. ABG showed pH, pCO_2_, and pO_2_ of 7.32, 39.3, and 40.5, respectively. CT head, CXR, and x-ray KUB imaging indicated no acute process. An arterial line was inserted, and the temperature management protocol was followed; nevertheless, the four-hour objective was not achieved. The cardiology consulted in the ED did not recommend percutaneous coronary intervention (PCI) but suggested an echo, urine toxicology, and monitoring troponin.

**Figure 1:**
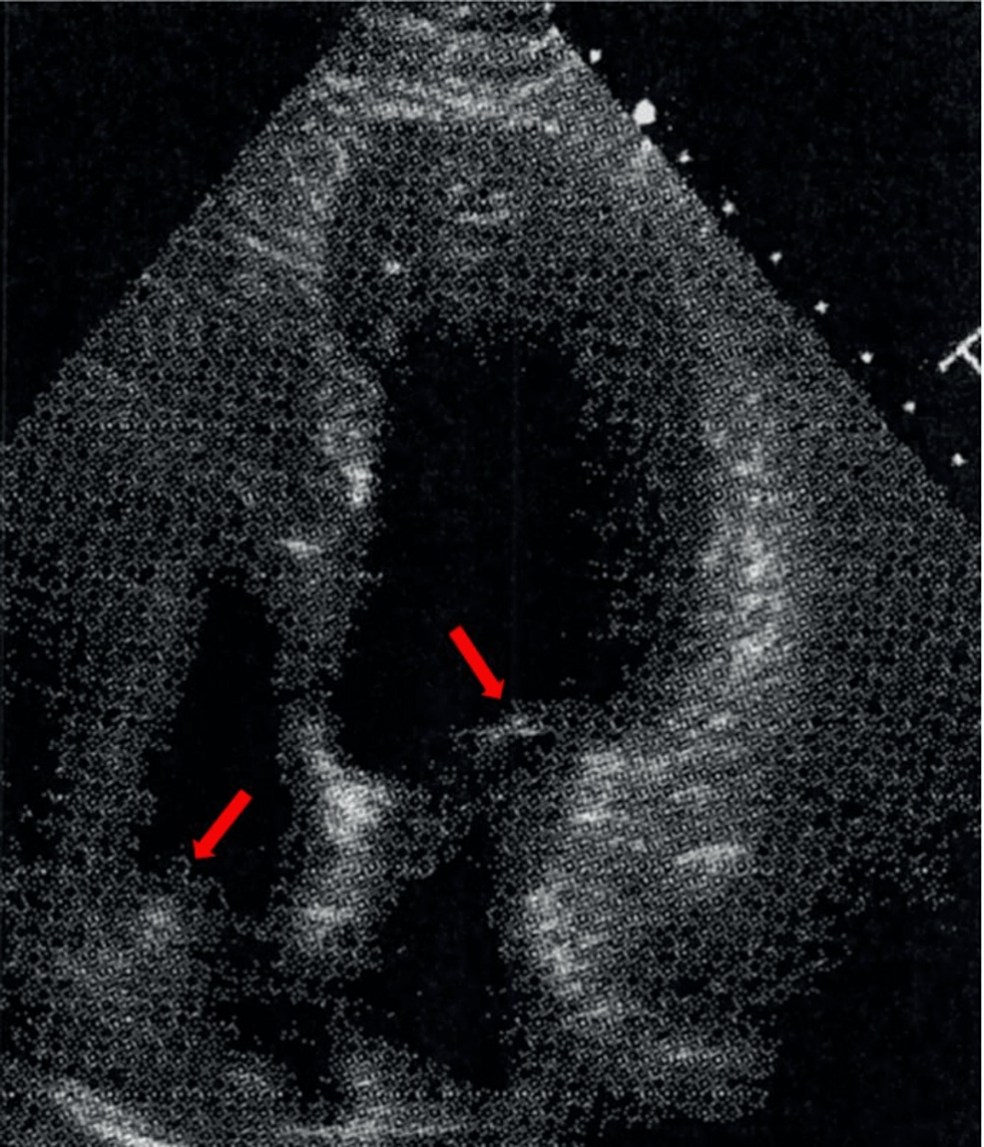
An echocardiogram image of the patient with mildly enlarged left ventricular (LV) wall thickness and pronounced trabeculation (shown in red arrows).

The patient was hemodynamically stable and transferred to the Intensive care unit. Cooling was optimized. Ventilation settings on arrival were respiratory rate 16, tidal volume (VT) 450, PEEP 5, FiO_2_ 40. The patient's initial laboratory studies and toxicology screen did not show any abnormalities. Her transthoracic echocardiography revealed a pseudo-normal LV filling pattern, concurrent aberrant relaxation, elevated filling pressure, and a mildly enlarged left ventricular wall thickness with pronounced trabeculation and left ventricular ejection fraction (LVEF) of 55%-60% (Figure 1).

The LV's spongiform appearance suggested non-compaction cardiomyopathy, which was thought to be a potential cause of cardiac arrest. Cardiac MRI was performed and confirmed the diagnosis of isolated LVNCC. Next, she was weaned from the ventilator and underwent ICD placement for secondary prevention of sudden cardiac death. On the 10th day of hospitalization, she was discharged home. Echocardiographic screening and genetic evaluation, including counseling and genetic testing of first-degree relatives, were also advised. After the cardiac arrest event, the patient was followed up in the cardiology clinic without any further events. However, the patient's recent device clinic check-up revealed several episodes of paroxysmal atrial fibrillation. With a CHA₂DS₂-VASc Score of 1 (female), we recommended starting apixaban 5 mg BID for stroke prevention and Metoprolol succinate 25 mg QD for rate control.

## Discussion

LVNC describes a complex myocardial disorder characterized by prominent Left Ventricular (LV) trabeculae, a thin compacted layer, and deep intertrabecular recesses [4]. LVNC was previously also called spongy myocardium or hypertrabeculation syndrome, but these terms should not be used interchangeably with LVNC [5]. LVNC had been classed as "unclassified" cardiomyopathy [6]. However, emerging evidence suggests that it should be reclassified as a distinct cardiomyopathy. Isolated LVNC occurs in the absence of other cardiac or noncardiac congenital abnormalities. LV non-compaction is a rare form of congenital cardiomyopathy and was first described in 1984 [7]. The prevalence among patients undergoing echocardiography is estimated at 0.01% to 1.3% [1,3,8].

From the fifth to the eighth week of development, the growing coronary vasculature from the epicardium invades the myocardium concurrently with LV trabecular compaction. This process, which coincides with myocardium development, may be triggered by hypoxia in the myocardium's outermost subepicardial layer [9]. From the heart's base to the apex and from the epicardium to the endocardium, LV compaction develops. Non-compaction Cardiomyopathy (NCM) is thought to be caused by a halt in trabecular compaction during this stage of embryogenesis. A two-layered myocardium comprises a compacted epicardial layer, and a non-compacted layer comprises a loose network of interwoven fibers, prominent trabeculations, and deep endomyocardial recesses that communicate with the LV cavity but not with the coronary circulation would result from the cessation of compaction. It has been hypothesized that the temporal variation of myocardial maturation failure might explain the wide range of pathological and clinical manifestations of NCM [10].

The clinical signs and symptoms of LVNC are variable and may include dyspnea, chest pain, palpitations, syncope, or an abnormal EKG or echocardiogram [3]. Obvious cardiac symptoms and signs may not be present. The primary complications of LVNC are Heart Failure (HF), atrial and ventricular arrhythmias, sudden cardiac arrest, and thromboembolic events, including stroke [3,11]. Syncope and seizures may be the initial signs of LVNC. Based on reports from some studies, syncope, and seizures appear to be warning indicators for LVNC patients at high risk for sudden death [12]. Malignant arrhythmias or sudden cardiac arrest is a rare presentation and constitutes only about 2% to 5% of cases of presentation [13].

LVNC can be either sporadic or familial. According to various statistics, 12% to 50% of individuals with LVNC have a family history of LVNC. [3] Autosomal dominant inheritance is more prevalent than X-linked or recessive inheritance [14]. The genetic loci associated with significant cardiomyopathies are increasingly recognized to overlap significantly. Different cardiomyopathic phenotypes have shared molecular etiology, including overlapping phenotypes between LVNC and Hypertrophic Cardiomyopathy (HCM) [15] and LVNC and apical HCM [16]. Despite the genetic overlap between LVNC and HCMs, LVNC co-occurs with congenital heart disease or Wolff-Parkinson-White syndrome more frequently than HCM.

A wide range of gene mutations has been found in LVNC patients, especially in genes coding for sarcomeric, cytoskeletal, Z-line, and mitochondrial proteins. Sarcomeric proteins are the most often mutated proteins (82%) [16] Variants in 66 genes in patients with LVNC were described in a systematic review of 561 patients from 17 studies [16], including genes encoding beta-myosin heavy chain (MYH7), titin (TTN), hyperpolarization-activated cyclic nucleotide-gated potassium channel 4 (HCN4), LIM domain binding protein 3 (LDB3), dystrobrevin (DTNA), tafazzin (TAZ), and lamin [17].

Annualized cardiovascular mortality of LVNC is 4% [18]. High morbidity and mortality rates in children and adults are associated with isolated LVNC. Patients with LVNC with HF or asymptomatic LV systolic dysfunction are treated according to guideline-directed medical therapy for HF [19].

Implantable cardioverter-defibrillator (ICD) treatment is essential for the secondary prevention of sudden cardiac arrest (SCA) in patients with LVNC cardiomyopathy. Patients with LVNC cardiomyopathy and atrial fibrillation should be anticoagulated based on the CHA2DS2-VASc scoring system. According to ACC/AHA 2008 Guidelines for Device-Based Therapy of Cardiac Rhythm Abnormalities, there is sufficient evidence to indicate that ICD treatment for patients with LVNC, an LVEF of 35%, and New York Heart Association (NYHA) class II to III HF; or the combination of LVNC, an LVEF of 35%, and positive family history for sudden cardiac arrest (Grade 2C) [20]. End-stage HF patients with LVNC are eligible for advanced therapy such as heart transplantation [3].

## Conclusions

Sudden cardiac arrest is an unusual presentation of LV non-compaction cardiomyopathy. LVNC can go undetected, resulting in sudden cardiac death as the initial presentation. A high level of clinical suspicion is required to diagnose LVNC. Also, because LVNC is associated with a higher risk of poor outcomes, prompt diagnosis and treatment are key. Patients with LVNC should be monitored closely to prevent further complications.
